# Cognitive Decline in Older People with Multiple Sclerosis—A Narrative Review of the Literature

**DOI:** 10.3390/geriatrics7030061

**Published:** 2022-06-05

**Authors:** Hsueh-Sheng Chiang, Alka Khera, Barbara E. Stopschinski, Olaf Stuve, John Hart, Brendan Kelley, Trung Nguyen

**Affiliations:** 1 Department of Neurology, University of Texas Southwestern Medical Center, Dallas, TX 75390, USA; alka.khera@utsouthwestern.edu (A.K.); barbara.stopschinski@utsouthwestern.edu (B.E.S.); olaf.stuve@utsouthwestern.edu (O.S.); jhart@utdallas.edu (J.H.J.); brendan.kelley@utsouthwestern.edu (B.K.); trungp.nguyen@utsouthwestern.edu (T.N.); 2 School of Behavioral and Brain Sciences, The University of Texas at Dallas, Richardson, TX 75080, USA; 3 VA North Texas Health Care System, Dallas, TX 75216, USA

**Keywords:** multiple sclerosis, cognitive aging, cognitive decline, neurodegeneration, dementia

## Abstract

Several important questions regarding cognitive aging and dementia in older people with multiple sclerosis (PwMS) are the focus of this narrative review: Do older PwMS have worse cognitive decline compared to older people without MS? Can older PwMS develop dementia or other neurodegenerative diseases such as Alzheimer’s disease (AD) that may be accelerated due to MS? Are there any potential biomarkers that can help to determine the etiology of cognitive decline in older PwMS? What are the neural and cellular bases of cognitive aging and neurodegeneration in MS? Current evidence suggests that cognitive impairment in MS is distinguishable from that due to other neurodegenerative diseases, although older PwMS may present with accelerated cognitive decline. While dementia is prevalent in PwMS, there is currently no consensus on defining it. Cerebrospinal fluid and imaging biomarkers have the potential to identify disease processes linked to MS and other comorbidities—such as AD and vascular disease—in older PwMS, although more research is required. In conclusion, one should be aware that multiple underlying pathologies can coexist in older PwMS and cause cognitive decline. Future basic and clinical research will need to consider these complex factors to better understand the underlying pathophysiology, and to improve diagnostic accuracy.

## 1. Introduction

Thanks to the advancement of disease-modifying therapy in multiple sclerosis (MS), and the resultant reduction in demyelinating lesions and neurologic sequelae, people with MS (PwMS) now have increasingly longer life expectancy, although it remains 6–10 years shorter than that of the general population [1,2]. Improved ability to accurately diagnose MS has increased our recognition that MS can also manifest after the age of 50 (i.e., late-onset MS), and may progress more rapidly in older individuals [3]. The net result of these factors is an increasing number of older PwMS in the US and globally. Cognitive changes related to the MS disease process have long been recognized [4], and continue to attract great research interest as a common clinical symptom in PwMS [5,6]. Nevertheless, in older PwMS, what contributes to the development of new cognitive symptoms or the progression of cognitive impairment remains a challenging question for both clinicians and researchers.

Case vignette: An older woman presents to the clinic with a long-standing diagnosis of MS and associated cognitive impairment that has been stable for years. However, she reports that now, at the age of 71 years, she has noticed more trouble remembering details of conversations, and she frequently repeats questions. Because of this, she missed her doctor’s appointments, and had to start using a pill organizer to manage her medications. Otherwise, there have not been any new or worsening neurological symptoms. A recent standardized neuropsychological assessment identified a decline to below-average performance in memory learning and retention in both verbal and non-verbal materials (i.e., California Verbal Learning Test and Rey–Osterrieth Complex Figure, respectively), as well as category (animal) fluency, representing a decline in her performance compared to five years prior, along with previously identified impairments in attention, executive function, and processing speed (i.e., Digit Span, Trail Making Test A and B, Letter Fluency, and Digit Symbol Modalities Test) (see a broad overview of these tests in [7]). She wonders if she is developing dementia, and whether there is any medicine that could help her memory. In this scenario, is there enough evidence to suggest an alternative diagnosis (such as Alzheimer’s disease (AD)) other than aging with MS? If so, what are the next steps to solidify a suspected diagnosis?

Although a well-established body of evidence has demonstrated prevalent cognitive deficits related to MS in attention, processing speed, and episodic memory in PwMS [4,5,8], as well as other cognitive phenotypes recognized more recently [6], it is still unclear how cognitive deficits may progress or change when PwMS become older. In this review article, we address the current evidence regarding the following questions: *Do older PwMS have worse cognitive decline compared to older people without MS? Can older PwMS develop dementia or other neurodegenerative diseases such as AD that may be accelerated due to MS? Are there any potential biomarkers that can help to determine the etiology of cognitive decline in older PwMS? What are the neural and cellular bases of cognitive aging and neurodegeneration in MS?* These questions are discussed in three main sections. In the first section, cognitive aging in MS and differentiation in cognitive profiles between MS and other diseases are addressed based on studies using standardized neuropsychological measures. In the second section, dementia in MS and its determinants (e.g., underlying pathologies and diagnostic examinations) are addressed. In the third section, studies looking at the neural and cellular bases of aging and neurodegeneration associated with MS are discussed. We then return to discuss the vignette, and conclude with summarized answers to the raised questions. We hope to provide a focused and critical narrative review of the current literature to improve the understanding of aging and cognitive changes amongst clinicians and researchers who work with older PwMS, and to generate ideas for future research.

## 2. Cognitive Aging and MS: Do Older PwMS Have Worse Cognitive Decline Compared to Older People without MS?

Cognitive impairment is more prevalent in older MS patients than younger PwMS. One study found that 77.4% of MS patients older than 55 years may demonstrate impairment in two or more cognitive domains—significantly greater than the figure of 42.8% in younger PwMS [9]. A Norwegian study found that 48% met their set criteria for cognitive impairment—defined as having an impaired score (1.5 standard deviations below the mean) in at least two of the four main cognitive domains (psychomotor speed, attention, learning/memory, and executive function)—after 30 years of MS disease duration [10]. Given the higher percentage of cognitive impairment in older PwMS, the question arises as to whether this cognitive impairment is primarily driven by the MS disease process, expected for aging in MS, or accelerated decline as a result of both MS and aging. One must also consider that the older are at risk of other causes of cognitive impairment, including AD, regardless of prior MS diagnosis.

Several studies have attempted to address the question of how cognition evolves over the lifespan in PwMS. Earlier research suggested that cognitive decline in MS progresses with age (particularly in processing speed and verbal learning [8,11]), though at the same pace and degree as age-related changes in healthy control (HC) subjects. This finding indicates the absence of accelerated cognitive decline in older PwMS, and argues against interaction between aging and MS. Based on 245 PwMS (18–74 years old) compared to 188 HC subjects on two measures of processing speed (the word reading and color naming trials of the Stroop Test), Bodling et al. (2009) [12] found that a decrease in processing speed occurred in both MS (patients compared to HCs) and aging (older compared to younger cohorts), but no interaction existed between these two factors. However, these measures of processing speed are less sensitive to the processing speed impairment typical of MS (compared to the Paced Auditory Serial Addition Test (PASAT) or Symbol Digit Modalities Test (SDMT), which require greater executive control and stress the frontal–subcortical tracts much more [5]). A study by Roy et al. (2017) [13] utilized the SDMT (for processing speed) and California Verbal Learning Test, Second Edition (CVLT-II; for verbal learning and memory). In this study, the degree of cognitive impairment in PwMS remained consistent across the lifespan. The authors concluded that the evidence of accelerated cognitive decline in older PwMS was absent, and such accelerated change may suggest other pathologies rather than aging in MS. One cited limitation of this study was a higher education level in the HC group compared to the MS group, although this hypothetically should have increased the ability to measure differences between the groups due to increased cognitive reserve (i.e., the presence of protective factors against cognitive decline, such as greater levels of education [14]) in the HC group. Based on these studies, researchers had previously concluded that the rate of cognitive change in PwMS does not accelerate with aging [2].

In contrast to prior research, more recent studies have shown evidence of interaction between aging and MS in older PwMS when different study designs were considered and more comprehensive measures were included in the analysis. Pagnotti et al. (2021) [15] compared cognitive functions in late-onset MS (LOMS; age of symptom onset > 40 years; mean 48.4) and adult-onset MS (AOMS; age of symptom onset up to 40 years; mean 28.8). LOMS patients demonstrated more impaired age-adjusted scores in visual memory and working memory compared to AOMS patients, after taking into account both disease duration and comorbid cardiac diseases. The authors posited that inflammatory changes associated with MS may be exacerbated in older PwMS due to age-related brain changes, and together these changes may overwhelm the available cognitive reserve, leading to a faster cognitive decline in LOMS patients. This study used a slightly different cognitive battery for AOMS versus LOMS. Although this is unlikely to have substantially impacted the results, it would be important to replicate these results using identical batteries in both groups. Another study [16] included 84 PwMS (30 young AOMS, 30 older AOMS, and 24 older LOMS) and 50 HC subjects (25 young, 25 older). By comparing young versus older subgroups across AOMS and HCs, the authors found an interaction effect between age and MS on attention, executive function, and processing speed performance, while such an interaction was not found in episodic memory measures. Tremblay et al. also found that longer disease duration resulted in more impaired processing speed and working memory [16]. Notably, this study was among the first to include psychosocial variables such as anxiety, depression, fatigue, and medical comorbidities that could confound the effects of MS on age. Despite the many merits of the above two studies, it is unclear whether the definition of symptom onset included cognitive symptoms in the initial presentation that led to an MS diagnosis, as has been more recognized in recent years [5], which may potentially have biased the results if such patients were unevenly distributed in AOMS versus LOMS. A few other studies have also suggested that older age plays a role in cognitive changes in MS. Ruano et al. (2017) [17] found that the presence of cognitive impairment (i.e., impaired scores in at least two cognitive domains) was associated with older age regardless of MS clinical subtypes. Baird et al. (2019) [18] found that processing speed was worse in older PwMS, while visuospatial learning and memory were worse in both older and middle-aged PwMS, when controlled for factors such as physical activity and years since diagnosis. Both Jakimovski et al. (2019) [19] and Roth et al. (2018) [20] confirmed that processing speed was impaired in older PwMS compared to similarly aged HCs—a finding that is well-established in young PwMS. Although Roy et al. (2017) [14] concluded that MS did not accelerate age-related cognitive changes, upon closer examination of their results, CVLT-II showed a marginally significant trend (*p* = 0.02, exceeding their threshold of 0.01), suggesting more rapid decline in verbal learning in MS due to older age.

One ought to keep in mind when reviewing these studies that cognitive reserve may also influence the trajectory of cognitive aging in MS, although most evidence thus far is based on younger rather than older PwMS. Cognitive reserve is a theoretical construct established in the aging and dementia literature [14]. It is used to explain some degree of individual variability in cognitive performance, and is thought to explain the observed association between certain protective factors (education, level of intellectual/leisure activity, employment status, vocabulary, etc.) and lower incidence and prevalence of cognitive decline. After controlling for brain atrophy, higher cognitive reserve (using education, premorbid IQ, and cognitive leisure activities) in MS is associated with better verbal memory and verbal fluency performance cross-sectionally, but not in attention or processing speed [21]. However, cognitive change over a two-year follow-up was explained not by cognitive reserve, but by the degree of brain volume and lesions. Another study showed that cognitive reserve (defined similarly as in [21]) and cortical atrophy together predicted the performance of verbal memory and processing speed, while older age and worsening cortical atrophy, rather than cognitive reserve, were predictive of cognitive deterioration over 1.6 years [22]. These studies suggest that greater cognitive reserve may be protective through better compensation for aging-related cognitive changes, but that the ability to compensate is eventually overwhelmed in the face of the disease’s progression. As opposed to the above studies showing cognitive reserve alone to not be predictive of subsequent cognitive decline, one study found that lower cognitive reserve (i.e., education, vocabulary) was predictive of greater cognitive decline in processing speed measures at the 5-year follow up [23]. However, this study did not include volumetric measures that could potentially confound the findings, as shown by other studies above. It remains unclear what role cognitive reserve plays in predicting cognitive decline in older PwMS with LOMS, or in older PwMS more generally. Answering this question could help us to identify PwMS who are at risk of cognitive decline, and whether efforts to increase cognitive reserve by introducing intellectual enrichment programs or other interventions could be helpful in protecting against cognitive decline [24].

To address more specifically how cognitive changes in older PwMS may differ from that in older adults with other neurodegenerative conditions, research was conducted to compare cognitive impairment associated with older PwMS as opposed to those with amnestic mild cognitive impairment (aMCI—a prodromal stage of AD) [25] or AD—the most common neurodegenerative disease in the older. Such studies can be quite informative for clinical management and research involving older PwMS. In the first study of this kind by Filley et al. (1989) [26], patients with progressive MS showed worse performance in attention and processing speed, but better performance in learning, memory, and verbal skills, than those diagnosed with AD. More recently, Roy et al. (2018) [27] examined older PwMS who were separated into cognitively impaired and unimpaired subgroups, in comparison to AD, aMCI, and HCs (*n* = 20 in each group). Despite no confirmatory biomarkers for AD diagnosis, they found that the impaired MS subgroup showed decreased category fluency similar to aMCI, but did not demonstrate as much rapid forgetting (measured by retention of verbal memory) as the AD and aMCI groups. They concluded that there was some overlap between MS and aMCI regarding their cognitive profiles, but that MS was distinctly different from AD. However, it is debatable whether impaired category fluency may represent emerging cognitive impairment due to other etiologies, as suggested by Jakimovski et al. (2019) [19], who also found impaired category fluency in older PwMS compared to similarly aged HCs. Other studies support the distinction between cognitive profiles in older MS and aMCI patients. Synthesizing the available studies comparing aMCI patients, older PwMS have (1) worse performance in processing speed, (2) better performance in measures of cued memory, picture naming, and executive function (after controlling for processing speed) [20], and (3) relatively preserved semantic autobiographical memory and memory storage (recognition) [28,29]. Overall, these studies indicate that certain neuropsychological measures, when impaired, should raise concern for AD-related pathologies in older PwMS.

Several important comorbidities of cognitive decline are highly prevalent in MS, which may contribute to the observed cognitive deficits, increased cognitive decline, and increased mortality—especially in older PwMS [30,31]. In particular, vascular comorbidities (hypertension, hyperlipidemia, ischemic heart disease, etc.) are more prevalent in MS compared to the general population, and the prevalence increases with age [30,32]. Very few studies on cognitive performance in older PwMS have accounted for these comorbidities thus far [15,16], so future research will need to consider the potential impact of these MS-related and age-related comorbidities on cognitive aging in MS.

In summary, despite heterogeneity in the samples and methodologies, recent studies suggest that older PwMS demonstrate more rapid decline in multiple cognitive domains—both in those typically observed in MS (e.g., attention, executive function, working memory, processing speed, visual memory), and in domains less typically impaired in MS (e.g., category fluency). There is also evidence that the pattern of cognitive impairment in older PwMS can be distinguished from that of aMCI and AD patients, especially in measures of semantic memory and memory storage/retention. The effects of cognitive reserve on this decline are not yet clear. In the future, study designs considering age-related and MS-related confounding factors (e.g., fatigue, cognitive reserve, depression, and vascular comorbidities), as well as disease duration and clinical subtypes (e.g., clinically isolated syndrome, relapsing–remitting MS, primary and secondary progressive MS), will be required to clarify this area of research. Prospective longitudinal studies are lacking, and should be conducted to replicate and expand upon the findings of cross-sectional studies. This could also aid clinical care through establishing age-adjusted normative data for cognitive performance in PwMS as a reference point to identify abnormal cognitive aging in older PwMS.

## 3. Dementia and MS: Can Older PwMS Develop Dementia or Other Neurodegenerative Diseases? Are There Any Potential Biomarkers That Can Help to Determine the Etiology of Cognitive Decline in Older PwMS?

While cognitive impairment and its contributions to functional impairment and disability in MS are widely recognized, dementia in MS has been less well-defined. Dementia describes a condition where cognitive abilities have become impaired to a degree that they interfere with daily activities. More specific criteria are available; for example, the diagnostic guidelines for AD [33] have established criteria for all-cause dementia to define clinical settings where cognitive or behavioral symptoms *“(1) interfere with the ability to function at work or usual activities, (2) represent a decline from previous levels of functioning and performing, and (3) are not explained by delirium or major psychiatric disorder”*, established based on history and cognitive assessment indicating impairment in at least two cognitive domains. This decline in the context of dementia should also be progressive over time. *The Diagnostic and Statistical Manual, Fifth Edition* (DSM-5) [34] utilizes the synonymous term Major Neurocognitive Disorder, with the criteria that there is evidence of cognitive decline from a previous level of performance in at least one cognitive domain based on concerns from the individual or others, as well as impairments in cognitive assessments that interfere with independence in everyday activities [34]. The DSM-5 provides more specific criteria for neurocognitive disorder subtypes (Alzheimer’s disease, frontotemporal, Lewy bodies, vascular, etc.) as well as major neurocognitive disorders due to other medical conditions, for which MS is the exemplar.

Estimates of the prevalence of dementia in MS range from 22 to 28% [35,36]. Acknowledging the variability in assessing cognitive impairment in MS, researchers recently proposed investigational criteria for neurocognitive disorders due to MS, combining the DSM-5 criteria for major neurocognitive disorder with a requirement of performance 1.5 standard deviations below the normative mean in two or more cognitive domains in neuropsychological testing [37]. Applying these criteria in an academic MS multidisciplinary clinic, 13.8% of patients met the research criteria for neurocognitive disorder due to MS, compared to 20.5% when using the established DSM-5 criteria alone [37]. These authors and others [38,39] have provided commentary regarding the controversies surrounding the diagnosis of dementia in MS, including societal stigma with the term “dementia” and the presumed association with neurodegenerative processes when used colloquially, leading to a reluctance in assigning a diagnosis of dementia to younger PwMS. There are also direct implications to having a diagnosis of dementia, including limitations on driving, the need for financial and medication oversight, diminished capacity for informed consent, etc. [38]. These have major implications for the next decades of a person’s life when diagnosed as demented in their younger or middle adulthood. These issues limit the investigation of dementia due to MS and, thus, the understanding of the development of other dementing illnesses in PwMS. For instance, a recent study reported on the higher risk for a diagnosis of both early- and late-onset dementia in PwMS based on administrative claims data, though acknowledged the limitation of attributing cognitive impairments to MS, AD, or other related dementias due to the diagnostic challenges [40].

Although it would be logical to conceptualize MS as an etiology of progressive cognitive impairment and dementia, relatively few studies outside of case reports have done so. Several case reports [41,42,43] have described patients with profound cognitive impairment and dementia along with clinical, radiographic, and neuropathological findings consistent with MS as the primary etiology, in the absence of pathological hallmarks of other neurodegenerative diseases. In these studies, common neuropathological findings include gross cerebral atrophy as well as corpus callosal and subcortical demyelinated lesions that can have predilections for certain brain regions, or can be widespread [41,42,43]. The pattern of deficits is often described as a “subcortical dementia”—with more impaired processing speed, memory retrieval, executive dysfunction, mood disturbances, and neurogenic personality disorder—in comparison to “cortical dementia”, showing more predominantly impaired memory retention, language, and visuospatial functions, such as in AD [26]. However, “cortical multiple sclerosis” is becoming increasingly recognized, as highlighted in cases with prominent cortical dysfunction and dementia without the typical history of sensory and motor symptoms [44,45]. This variability in patterns of cognitive deficits can interfere with the determination of the etiology of dementia in patients with MS if based solely on neuropsychological performance.

The greatest amount of research into neurodegenerative diseases concomitant with MS has been on AD. In their review of 45 autopsy cases of PwMS, Dal-Bianco et al. reported that pathological signs of AD—namely, amyloid plaques and neurofibrillary tangles—were found at an incident rate similar to that seen in the normal aging population, and thus concluded that the chronic inflammatory state of MS does not impact the pathogenesis of AD [46]. Another autopsy study [47] came to similar conclusions after analyzing the inflammatory cell infiltrates in 67 MS autopsies from different disease stages and 28 control patients without known neurological disease. They found that neurodegeneration continued to progress, and exceeded the levels in controls only when there were coexisting age-related pathologies from AD and vascular disease, while neuroinflammation declined to levels comparable to controls in older patients. The authors concluded that the MS-related disease process may cease in older PwMS with long-standing disease, and that other pathologies—including AD and vascular disease—become the primary drivers of neurodegeneration in older individuals. Similar reports of cases with MS and AD were reviewed and summarized by Luczynski et al. (2019) [48].

The approach to diagnosing AD has evolved over time, and the most recent research framework incorporates biomarkers for amyloid β (Aβ), tau, and neurodegeneration into the AT(N) system [49]. While assessing AD biomarkers may not always be clinically feasible, it can be effective in providing diagnostic clarity regarding the coexistence of MS and AD, as illustrated in the case series reported by Flanagan et al. (2014) [50]. These AD biomarkers include cerebrospinal fluid (CSF levels of amyloid β 42, total tau, and phosphorylated tau), fluorodeoxyglucose positron emission tomography (FDG-PET), and amyloid PET. However, assessing AD biomarkers in PwMS is not without caveats, as similar findings can be seen in MS [51,52,53,54]. For example, reduced CSF Aβ is found in MS, and is associated with worse disease progression, although this is hypothesized to be due to MS-related white matter pathology, as white matter demyelination correlates with reduced uptake in amyloid PET [55]. Further studies with direct comparisons of biomarkers between PwMS and/or AD are warranted to clarify their interpretation.

However, a potential interaction between the pathologies of MS and AD has been revealed by in vivo data obtained via PET imaging techniques. Zeydan et al. demonstrated reduced Aβ binding on Pittsburgh compound B (PiB) PET imaging in areas of white matter hyperintensities than in normal-appearing white matter in older PwMS [56]. Interestingly, reduced PiB uptake in PwMS was associated with decreased visuospatial performance, and was proposed as a marker of large network integrity. In a second study, Zeydan et al. showed lower overall cortical Aβ deposition in PwMS compared to age-matched HC subjects, suggesting that the pathology of MS may prevent or slow down the age-related deposition of Aβ [57]. In contrast, tau-PET imaging (using the radiotracer ^18^F-flortaucipir, or AV-1451) did not reveal a difference between PwMS and HCs. These findings support the hypothesis that MS-specific inflammatory processes—such as via microglial activation—may facilitate the clearance of and reduction in Aβ protein deposition, while tau pathology may progress independently from amyloid deposition in older PwMS. However, others have hypothesized that decreased Aβ protein binding in MS can be linked to demyelination resulting from white matter injury, or to reduced remyelination with older age [55]. As previously discussed, in opposition to the PET data, neuropathological examination of older MS brains has so far not provided clear evidence of a potential interaction between the pathology of MS and other neurodegenerative conditions [46,47]. Nevertheless, some preliminary data have shown the presence of (1) soluble amyloid oligomers in MS brain homogenates and CSF samples [58], and (2) hyperphosphorylated tau and accumulation of insoluble tau in chronic experimental autoimmune encephalomyelitis in mice and secondary progressive MS in humans [59]. These findings suggest a role of amyloid and tau proteins in the pathogenesis of MS and neurodegeneration, and vice versa. Future studies integrating both ante-mortem cellular and imaging biomarkers as well as post-mortem neuropathology will help to reconcile these potential discrepancies and elucidate any underlying pathophysiology that may be shared between MS and AD in the context of older PwMS.

Vascular dementia (VaD) and dementia with Lewy bodies (DLB) are among the most common causes of dementia in the older, after Alzheimer’s disease [60,61]. The definition of VaD and vascular cognitive impairment (VCI) has evolved over time [34,62,63,64,65,66], though overall requires that the cognitive impairment be temporally related to strokes or be accompanied by significant neuroimaging evidence of cerebrovascular disease. PwMS have an increased risk of stroke [67,68], and would thus be more susceptible to developing VCI in addition to MS-related cognitive decline. Among the subtypes of vascular dementia, subcortical vascular dementia due to small-vessel disease [69] can present with progressive fronto-subcortical dysfunction (e.g., slowed psychomotor speed, impaired executive function, and impaired memory recall, but preserved recognition), similar to subcortical dementia as a result of MS [70]. Structural MRI showing multiple lacunar infarcts and microbleeds in the subcortical areas can help to distinguish the primary vascular cause from MS, thereby informing the underlying etiology. However, white matter changes (e.g., periventricular hyperintensities) due to small-vessel disease may be mistaken for MS demyelination if not cautiously interpreted, leading to misdiagnosis of MS [71,72]; on the other hand, confluent demyelinating lesions due to MS can resemble the appearance of small-vessel ischemic disease [72]. Careful examination of MRI findings integrated with the patient’s history and demographics can improve diagnostic accuracy to distinguish between vascular and MS lesions as the primary cause of cognitive decline [73]. DLB is characterized by both cortical and subcortical cognitive impairments [74,75]. Although logically the Lewy body pathology, just as that of AD, should coexist in older PwMS, this topic is understudied, and there have been no reported cases of MS with concomitant DLB. However, DLB’s core clinical features of parkinsonism [76], visual hallucinations [77,78], and rapid eye movement (REM) sleep behavior disorder [79,80] have also been described in MS, and may increase the diagnostic uncertainty of DLB in PwMS. Cognitive fluctuation is the core clinical feature of DLB that is the most difficult to assess [81], and has not been well-characterized in MS. Future research will be needed to better characterize the clinical DLB phenotype in MS, and to examine how coexisting Lewy body pathology (i.e., alpha synuclein) may interact with MS to potentially contribute to cognitive and neurologic decline in older PwMS.

Treatment options for cognitive impairment and dementia in MS are limited. Medications typically used for AD and DLB include cholinesterase inhibitors (e.g., donepezil, rivastigmine, galantamine) and the N-methyl-D-aspartate receptor antagonist memantine. Unfortunately, these agents do not appear to be effective for treating cognitive impairment in MS [82,83], and some studies have suggested that memantine may even worsen MS-related symptoms [84,85,86]. Whether these treatment options would be safe and effective in patients with dementia due to MS or with concomitant dementia syndromes remains to be seen.

To summarize, the evaluation of dementia in older PwMS can be complicated, as dementia due to MS is not systematically studied and defined, and there can be coexisting neurodegenerative diseases. Reported case studies may suffer from selection bias, and may not be representative of older PwMS in general. Hence, larger-scale studies are warranted to systematically examine established biomarkers and neuropathological findings in older PwMS with or without dementia, and to establish the associations between clinical presentation, diagnostic testing, and pathology.

## 4. What Are the Neural and Cellular Bases of Cognitive Aging and Neurodegeneration in MS?

So far, we have addressed cognitive changes associated with aging and dementia in PwMS. On the molecular and cellular levels, multiple mechanisms have been proposed to contribute to cognitive decline in PwMS. Some reports have shown that aging may reduce the ability of older MS patients’ brains to recover from inflammatory attacks, because of reduced neuroplasticity due to impaired synaptic functions (e.g., inflammatory synaptopathy) affecting long-term potentiation and depression, which are strongly associated with mechanisms of learning and memory [87]. In addition, neuronal senescence has been discussed as a possible mechanism driving the progression of neurodegeneration in PwMS by promoting sustained and chronic inflammation, alteration of glial function with failure of remyelination and cellular recovery, and impairment of the blood–brain barrier’s integrity [88]. A recent publication has identified the toxic accumulation of the synaptic protein Bassoon in neurons as a potential driver of neurodegeneration (i.e., proteinopathy) in MS [89], although this is yet to be replicated in other human studies. A detailed discussion of these and other mechanisms is beyond the scope of this review, and can be found elsewhere [2,90,91]. Here, we seek to discuss current evidence derived from research using biomarkers including the CSF and structural magnetic resonance imaging (MRI), as well as their implications for cognitive aging and neurodegeneration in older PwMS.

Studies using CSF analysis have assessed the potential utility of some biomarkers to detect cognitive decline and/or neurodegeneration in PwMS. A recent meta-analysis of 64 articles and including >4000 study individuals revealed that markers of axonal damage (including neurofilament light chain (NfL) and total tau) and glial activation (including glial fibrillary acid protein (GFAP) and s100B) were higher in PwMS, as well as in patients with clinically isolated syndrome, compared to control patients. GFAP levels were higher in progressive MS compared to relapsing–remitting MS, while all other markers did not differ between MS subtypes [52]. Of note, no difference was found between the relapse and remission stages of MS in these markers except for NfL, although information was limited regarding how close in time CSF was sampled relative to the clinical event among the studies included in this meta-analysis. Even though the meta-analysis did not assess the association of these markers with cognitive performance, some studies have shown that CSF total tau is associated with disability and cognitive impairment [53,54]. In addition to markers of neurodegeneration, inflammatory markers have been also examined, and found to correlate with cognitive impairment in MS [92,93]. How aging and other age-related neurodegenerative processes may play a role in shifting neurodegenerative or inflammatory profiles in the CSF is less clear, and has only been examined by a few [94].

Imaging studies offer a valuable approach to examine in vivo how changes in gray and white matter may contribute to cognitive decline in older PwMS. Particularly, reduced volume in the thalamus and other subcortical gray matter structures based on MRI has been associated with both aging and neurodegenerative processes in MS. Hasan et al. (2011) [95] identified thalamic volume loss in a large cohort of PwMS (*n* = 109, age range 20.8–68.5 years) as a marker for disability compared to HC subjects, after adjusting for natural aging and whole-brain lesion volume. The thalamic atrophy occurred independently from lesions in this area, suggesting a neurodegenerative process as the underlying pathophysiology. Jakimovski et al. (2020) [96] examined a group of 112 older PwMS (mean age 60.3 years), and found that PwMS had reduced deep gray matter and the lowest thalamic volume compared to patients with Parkinson’s disease, AD, and aMCI. Interestingly, no difference in whole-brain volume loss was noted between patients with MS and other patient groups [96]. To further characterize and distinguish MS-related from age-related regional volumetric changes, a longitudinal study included 520 patients with relapsing–remitting MS and 130 HC subjects [97]. The authors found that the rate of global atrophy increased by 0.11% per decade from age 30 to 60 years in normal aging, while it decreased by 0.09% per decade in MS. Thalamic atrophy followed a similar pattern, increasing by 0.16% per decade from age 30 to 60 years in normal aging, while it decreased by 0.18% per decade in MS. Normal aging and MS-specific atrophy in the putamen and caudate did not vary by age. Notably, these subregions were not pre-selected, but instead ranked as the top 5 regions (among all 83 regions) using a data-driven approach. These results suggest that age-related thalamic changes may be more prominent than MS-specific changes in older PwMS. Although thalamic atrophy has been associated with cognitive performance [98], and is predictive of cognitive decline [99] in younger PwMS, how these dynamic volumetric changes in the thalamus and other brain regions may contribute to either the degree or the pattern of cognitive decline in older PwMS remains to be investigated.

The bulk of studies to date have concluded that white matter lesion burden evident on clinical MRI shows little correlation with the degree of cognitive impairment, compared to damage in gray matter and disruption in normal-appearing white matter [100,101]. More advanced MRI techniques have demonstrated decreased white matter integrity in MS in the corpus callosum, hippocampal and thalamic tracts, and other major white matter tracts [95,102,103]. Neuropathological studies support these in vivo imaging findings, demonstrating thalamic and hippocampal demyelination in MS [42,104,105]. Compared to research focused on gray matter volume, it is less clear how age may interact with MS-related white matter lesions/changes and contribute to cognitive changes in older PwMS.

Vascular risk factors are associated with brain atrophy and white matter disease in MS [106]. Drivers of cerebrovascular injury are more prevalent in PwMS, and the prevalence of these conditions further increases with age [30,32]. Thus, studies seeking to analyze CSF and imaging biomarkers will need to consider both age and vascular diseases to better disentangle their impact on the neural basis of cognitive decline and neurodegeneration in older PwMS.

These findings suggest that cognitive decline in older PwMS is multifactorial, with both neuroinflammatory and neurodegenerative processes as important contributing factors. Studies using in vivo biomarkers (i.e., CSF markers for neurodegeneration and neuroinflammation; imaging markers for gray and white matter changes and other neurodegenerative processes) have promise to elucidate the underlying pathophysiology as well as identify an active window for more targeted intervention and disease monitoring.

## 5. Conclusions

We conclude first by going back to the initial questions: *Do older PwMS have worse cognitive decline compared to older people without MS?* Current evidence suggests that aging interacts with the MS disease process, and more recent studies better account for confounding MS-related symptoms (fatigue, disease duration, depression, etc.) and comorbidities such as cardiac disease. Cross-sectional work suggests that cognitive profiles of aging and dementia due to MS in older adults can be distinguished from those in other neurodegenerative diseases, except in the case of “cortical MS”. *Can older PwMS develop dementia or other neurodegenerative diseases such as AD that may be accelerated due to MS? Are there any potential biomarkers that can help to determine the etiology of cognitive decline in older PwMS?* Diagnosis of dementia in older PwMS continues to pose high diagnostic uncertainty given the overlapping phenotypes associated with variable pathologies that can only be confirmed by post-mortem autopsy. Detecting other neurodegenerative diseases (e.g., AD) based on standardized biomarkers may be useful in making a diagnosis, but how best to interpret these biomarkers in PwMS remains uncertain. The sensitivity and specificity of the currently available tests to detect underlying pathologies in MS (e.g., Aβ and tau levels in CSF, and amyloid PET) remain yet to be determined. Post-mortem neuropathological studies did not support an accelerated AD pathology in chronic MS, but emerging evidence introduces contradicting views that MS may interact with (accelerate versus decelerate) AD pathogenesis. *What are the neural and cellular bases of cognitive aging and neurodegeneration in MS?* Multiple pathologies can coexist in the brains of PwMS, with complicated interactions involving neurodegeneration, neuroinflammation, and neurovascular mechanisms. CSF analyses to detect neurodegeneration and neuroinflammation, along with neuroimaging studies that detect regional gray and white matter changes as well as proteinopathies, hold promise to better elucidate the potential pathophysiology of age-related versus MS-related changes that often coexist in older PwMS.

Now, we come back to the vignette: The patient mentioned earlier has typical MS-related cognitive changes in attention, executive function, and processing speed. However, new-onset decline in memory retention and category fluency may be a warning sign of other disease processes—especially AD. Further studies are warranted to better answer the patient’s question. Structural brain MRI can help to evaluate the progression of regional atrophy (such as in the mesial temporal lobe), which may be more suggestive of AD, although MS may also cause cortical lesions and hippocampal demyelination, leading to atrophy. Small-vessel disease can also cause atrophy and white matter changes in older PwMS, and contributes to cognitive decline. Even though atrophy in particular structures—such as the thalamus—is linked to the MS disease process, it is not yet clinically applicable for making individual diagnosis and prognosis. To assist with diagnosis, CSF analysis for AD biomarkers versus amyloid PET can be considered to assess for comorbid AD pathology that can coexist with MS, with the caveat that the MS disease process may also interact with Aβ and tau, thus affecting the interpretation of the test results. Eventually, a good understanding of the existing diagnostic uncertainty and careful interpretation of the data will be necessary to make a well-formed clinical judgment. One should closely involve the patient in the thought process, with an open discussion if a potential treatment for a suspected diagnosis is considered.

## 6. Future Directions

As PwMS are living longer, it is important that more research efforts be directed to better understand how these processes contribute to cognitive decline in older PwMS. Several future directions are suggested and summarized here. To better characterize age-related versus MS-related cognitive changes and their potential interaction (e.g., accelerated aging in MS), study designs will need to consider potential confounding variables, disease duration, and clinical MS subtypes. Prospective longitudinal studies are needed to characterize the cognitive trajectory and replicate findings from cross-sectional studies to address how cognitive changes evolve as younger PwMS age. Establishing age-adjusted normative data for cognitive performance in PwMS will guide and serve as a reference for clinicians in the early detection of abnormal cognitive aging and consideration of alternative treatment in older PwMS. More systematic research is warranted to better define dementia and its cognitive/functional trajectory in older PwMS, combined with the examination of biomarkers and neuropathological correlates to establish the associations between clinical presentation, diagnostic testing, and pathology. Future research will need to investigate how MS and other coexisting age-related pathologies (such as AD, Lewy body disease, and cerebrovascular disease) may contribute to different patterns or progression of cognitive impairment. This research will benefit from using multimodal methodologies to better elucidate the pathophysiology at the cellular, systemic, and behavioral levels of cognitive aging and neurodegeneration in MS. The expected results will have significant clinical impact on the care of older PwMS by better measuring the progression of coexisting diseases, developing targeted therapies, and monitoring therapeutic responses.

## Data Availability

Not applicable.
